# Diurnal Control of Sensory Axon Growth and Shedding in the Mouse Cornea

**DOI:** 10.1167/iovs.61.11.1

**Published:** 2020-09-01

**Authors:** Sonali Pal-Ghosh, Gauri Tadvalkar, Beverly A. Karpinski, Mary Ann Stepp

**Affiliations:** 1 Department of Anatomy and Cell Biology, The George Washington University School of Medicine and Health Sciences, Washington, District of Columbia, United States; 2 Department of Ophthalmology, The George Washington University School of Medicine and Health Sciences, Washington, District of Columbia, United States

**Keywords:** cornea, sensory nerves, circadian rhythm, mice

## Abstract

**Purpose:**

The circadian clock plays an important role in the expression and regulation of various genes and cellular processes in the body. Here, we study diurnal regulation of the growth and shedding of the sensory axons in the mouse cornea.

**Methods:**

Male and female BALB/cN mice were euthanized 90 minutes before and after the lights are turned on and off; at 5:30 AM, 8:30 AM, 5:30 PM, and 8:30 PM. Nerve terminal growth, shedding and overall axon density were assessed at these four time points using confocal imaging after staining axons in en face whole mount corneas with antibodies against βIII tubulin, GAP43, and L1CAM. In addition, corneal epithelial cell proliferation, thickness, and desquamation were assessed using ki67, LAMP1, Involucrin, and ZO1.

**Results:**

Nerve terminal shedding took place between 5:30 AM and 8:30 AM and correlated positively with the timing of apical cell desquamation. After shedding the tips of the nerve terminals, axonal growth increased as indicated by increased axonal GAP43 expression. At 5:30 PM and 8:30 PM before and after the lights are turned off, cell proliferation was reduced, and epithelial thickness was maximal.

**Conclusions:**

Intraepithelial corneal nerve growth and shedding are under diurnal control regulated by the time of day and whether lights are on or off. Axons extend during the day and are shed within 90 minutes after lights are turned on. The data presented in this article shed light on the potential role that circadian clock plays in corneal pain and discomfort.

The impact of time of day and exposure to light on corneal epithelial cells has been studied in mice for decades. In the 1970s and early 1980s, studies showing diurnal control of corneal epithelial cell proliferation were published.^1^^–^^4^ These events were confirmed to be under circadian regulation; in that they persisted when mice were maintained in the dark. In 1994, Sandvig and colleagues^5^ assessed corneal epithelial migration and proliferation as a function of time after following injury in rats. Their results have been confirmed and extended by numerous groups.^6^^–^^10^ When maintained in a 12-hour-on/12-hour-off light cycle, corneal epithelial cells proliferate at a higher rate and migrate faster after injury while lights are on.^10^ Using mice expressing luciferase under the control of the circadian clock gene Period (PER::LUC mice), corneal epithelial cells have been shown to maintain circadian regulation of expression of clock genes in vitro.^6^^,^^8^^,^^9^ A recent study isolated RNA from mouse corneas every three hours for 24 hours and performed RNAseq analyses that reveal that 25% of the transcriptome is regulated over time.^11^ We hypothesize that corneal sensory axonal growth is also under diurnal control and that the apical tips of the intraepithelial corneal nerve terminals (ICNTs) are shed when lights are turned on. If so, this would be similar to what has been reported for the shedding of rod and cone outer segments.^12^^–^^14^

The term intraepithelial corneal nerves (ICNs) has been proposed to refer to the sensory nerves that innervate the mouse cornea.^15^^,^^16^ The thicker and more dense axons are found within the epithelial basal cell layer above the epithelial basement membrane where they have been referred to as subbasal nerves, but a more anatomically correct term is intraepithelial corneal basal nerves (ICBNs). The ICBNs branch apically and extend between epithelial cells towards the apical squames forming ICNTs. Recent three-dimensional (3D) FIBSEM imaging studies have shown that the ICNs and ICNTs are wrapped in epithelial cell membranes within the corneal epithelium.^17^ Together, the ICBNs and ICNTs comprise the ICNs.

To determine whether the ICNTs are shed in a diurnal manner, we euthanized mice 90 minutes before and 90 minutes after lights are turned on and off. We assessed nerve terminal apical extension and corneal epithelial cell desquamation using 3D confocal images after staining with antibodies against axonal proteins to visualize elongating ICNTs. In addition, the tight junction protein ZO1, the terminal epithelial differentiation marker involucrin (IVL), and the lysosomal protein and autophagy marker LAMP1 were assessed to determine whether changes in ICNT length were associated with corneal epithelial cell terminal differentiation and desquamation. Changes in overall ICN axon density, epithelial thickness, and epithelial cell proliferation were also observed as a function of time after lights are turned on and off.

## Materials and Methods

### Animals

All studies performed comply with The George Washington University Medical Center Institutional Animal Care and Use Committee guidelines and with the ARVO Statement for the Use of Animals in Ophthalmic and Vision Research. For these studies, seven- to eight-week-old male and female BALB/cN mice were used. Mice were euthanized at four different time points: 5:30 AM (ZT 22.5), 8:30 AM (ZT 1.5), 5:30 PM (ZT 10.5), and 8:30 PM (ZT 13.5). These time points represent sacrificing mice 90 minutes before and after the lights are turned on and off following the Zeitgeber time (ZT) scale.

### Fixation and Whole Mount Staining

All eyes were fixed immediately after enucleation in a paraformaldehyde-containing fixative (1× phosphate-buffered saline solution (PBS), 1% formaldehyde, 2 mmol/L MgCl_2_, 5 mmol/L ethylene glycol-bis(β-aminoethylether)-N,N,N′,N′-tetraacetic acid (EGTA), 0.02% NP-40) for 1 hour and 15 minutes at 4˚C, followed by two washes for 10 minutes each in 1× PBS containing 0.02% NP40 at room temperature. Tissues were then placed in 4:1 methanol: dimethyl sulfoxide for two hours at −20°C and then stored in 100% methanol at −20°C until used for whole-mount staining studies. The back of the eye was cut, and the retina, lens, and iris were removed before staining. Tissues were transferred to a graded Methanol-TritonX-100 series (75%, 50%, and 25% methanol:TritonX-100 for 15, 15, and 10 minutes, respectively). All incubations were performed with gentle shaking and at room temperature, unless otherwise specified. The eyes were washed twice in PBS, for 30 minutes each, followed by incubation with blocking buffer for two hours. Blocking buffer was made as follows: to 100 mL 1× PBS, 1 g of bovine serum albumin was added, the mixture was stirred for 10 minutes, 1 mL of horse serum was added, and the mixture was stirred for an additional minute. The tissues were incubated overnight with primary antibody diluted in blocking buffer at 4°C. The following antibodies were used: βIII tubulin (TUJ1; no. 801201; BioLegend, San Diego, CA, USA), mLAMP1 (no. AF4320; R&D Systems, Minneapolis, MN, USA), ki67 (no. ab16667; Abcam, Cambridge, MA, USA), GAP43 (no. NB300-143; Novus Biologicals, Littleton, CO, USA), L1CAM (no. MAB5272; Millipore, Burlington, MA, USA), ZO-1 (no. sc-33725; Santa Cruz Biotechnology, Dallas, TX, USA), and Involucrin (no. 924401; BioLegend). Appropriate secondary DyLite 488, 594, and 647 antibodies from Jackson Immunobiologicals (West Grove, PA, USA) were used for immunolabeling. The next day, the tissues were washed five times with PBS and 0.02% Tween 20 (PBST) for one hour each, blocked for two hours, and then incubated with secondary antibody diluted in blocking buffer overnight at 4°C. The following day, eyes were washed three times with PBST for one hour each, followed by nuclear staining with 4,6-diamidino-2- phenylindole (DAPI) for five minutes, and washed with distilled water. To achieve the best flattening, the corneas were placed epithelial side-up with mounting media (no. 17984-25, Fluoromount G; Electron Microscopy Sciences, Hatfield, PA, USA) and coverslipped.

### Confocal Microscopy and Image Analysis

Confocal microscopy was performed at the GW Nanofabrication and Imaging Center at the George Washington University Medical Center. A confocal laser-scanning microscope (Zeiss 710; Carl Zeiss, Inc., White Plains, NY, USA) was used to image the localization of Alexa Fluor 488 (argon laser; 488-nm laser line excitation; 495/562 emission filter; Jackson Immunobiologicals), Alexa Fluor 594 (561 diode laser; 594-nm nm laser line excitation; 601/649 emission filter; Jackson Immunobiologicals), and Alexa Fluor 647 (633 Diode laser; 647-nm laser line excitation; 671/759 emission filter; Jackson Immunobiologicals). Optical sections (z = 0.5 µm) were acquired sequentially with a 63× objective lens. The 3D images were rotated to generate cross-section views using Volocity software (version 6.3; Perkin Elmer, New York, NY, USA). High-resolution images were presented either as cross-sections projected through the length of the acquired image (135 µm), or as cross-sections projected through 0.5 µm of tissue. Each image subjected to quantification was obtained using the same confocal laser settings and the same intensity settings in Volocity to permit valid comparisons.

For Sholl analysis, images were acquired using the Zeiss Cell Observer Z1 spinning disk confocal microscope (Carl Zeiss, Inc.), equipped with ASI MS-2000 (Applied Scientific Instrumentation, Eugene, OR, USA) scanning stage with z-galvo motor, and Yokogawa CSU-X1 spinning disk. A multi-immersion 25×/0.8 objective lens, LCI Plan-Neofluor, was used for imaging, with oil immersion. Evolve Delta (Photometrics, Tucson, AZ, USA) 512 × 512 EM-CCD camera was used as detector (80-msec exposure time). A diode laser emitting at 568 nm was used for excitation (54% power). Zen Blue software (Carl Zeiss, Inc.) was used to acquire the images, fuse the adjacent tiles, and produce maximum intensity projections. The adjacent image tiles were captured with overlap to ensure proper tiling. All images were acquired using the same intensity settings. Sholl analysis was performed using ImageJ as described previously.^18^

### Cell Proliferation Studies

For cell proliferation studies, images were acquired on the Nikon E600 Fluorescent Microscope (Photometrics, Tucson, AZ, USA) and the numbers of ki67+ cells per field were analyzed using ImageJ. Two fields were imaged in each peripheral zone (four zones) and two images at the corneal center, so 10 fields total per eye were assessed.

### Sum Intensity Studies

The 3D en face images of apical, middle and basal regions of the confocal stack were generated using the XYZ plane view option in Volocity. Sum intensity for each color for the middle and basal section was obtained using ROI statistics in the Nikon Analysis Software (NIS-Elements AR Analysis 5.20.01).

### Pixel Intensity Studies

Cross-section images were generated using Volocity. In image J, the line tool was used to draw a line at the apical and mid region of the ICNTs. Select Analyze- Plot Profile. The numbers obtained were then analyzed using Prism.

### Measurement of ICNTs

The 3D en face images of apical, middle and basal regions of the confocal stack were generated using the XYZ plane view option in Volocity. The apical section was used for the measurement of ICNTs using the measuring tool in ImageJ.

### Statistical Analyses

Quantitative data are presented as mean ± standard error of the mean. All data were analyzed using one-way analysis of variance (ANOVA). All statistical tests were performed using the GraphPad Prism Program, Version 6 (GraphPad Software, Inc. San Diego, CA, USA). A *P* value < 0.05 was considered statistically significant.

## Results

### The Intraepithelial Corneal Nerve Terminals (ICNTs) Grow While the Lights are on

Studies of circadian rhythms typically refer to the Zeitgeber time (ZT) scale. The ZT scale goes from 0 to 23; 0 refers to when lights are turned on and 12 to when lights are turned off. At the GW animal facility mice are on a 12 hours on/12 hours off time schedule with lights going on and off at 7:00 AM and 7:00 PM, respectively. To determine the impact of the light cycle on the ICNs, we sacrificed mice 90 minutes before and 90 minutes after lights went on and off (ZT 22.5–5:30 AM, ZT 1.5–8:30 AM, ZT 10.5–5:30 PM, and ZT 13.5–8:30 PM) and processed their corneas for whole mount 3D confocal imaging.

βIII tubulin, GAP43, and L1CAM are expressed on the ICNs. While βIII tubulin and GAP43 are both considered regeneration associated proteins whose expression is greater in actively growing axons,^19^ GAP43 expression is more tightly linked to axon growth.^20^^–^^23^ If there are changes in axon tip shedding over time, corneal epithelial cells would need to upregulate phagocytosis and degradation of axonal debris within lysosomes. LAMP1 is a lysosomal marker ^24^ and L1CAM is a cell adhesion protein expressed on axonal membranes^25^^,^^26^ and on corneal epithelial basal cells.^27^

**Figure 1.**
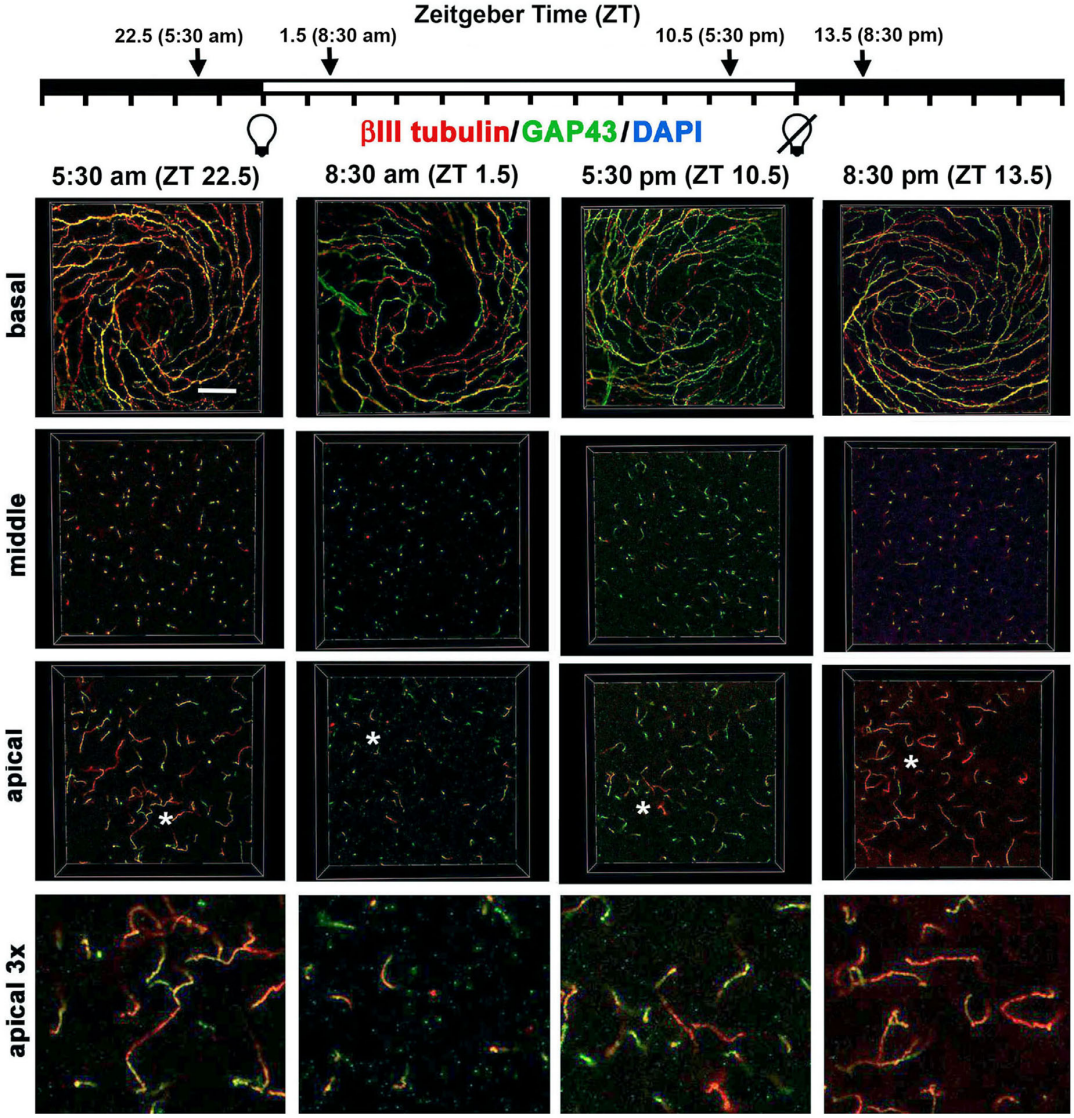
Localization of axonal proteins βIII tubulin and GAP43 in the mouse ICBNs and ICNTs visualized using 3D confocal imaging as a function of the time mice are euthanized. Six corneas from three male and three female mice euthanized at 5:30 AM (ZT 22.5), 8:30 AM (ZT 1.5), 5:30 PM (ZT 10.5), and 8:30 PM (ZT 13.5) were processed to visualize βIII tubulin and GAP43. Representative confocal images from a male cornea were acquired at the basal aspect of the corneal epithelium to show the ICBNs, at the middle of the corneal epithelium showing ICNTs perpendicular to the basal surface that appear as puncta, and the apical aspect of the cornea where many of the ICNTs turn and extend parallel to the ocular surface forming parallel ICNTs (pICNTs). The regions indicated by the *asterisks* in the apical images were magnified × 3 and are presented at the far right to highlight the pICNTs and their loss between ZT 22.5 and ZT 1.5. The localization of GAP43 and βIII tubulin varies within the axons; these data are quantified in Figure 3. *Scale bar:* 25 µm.

**Figure 2.**
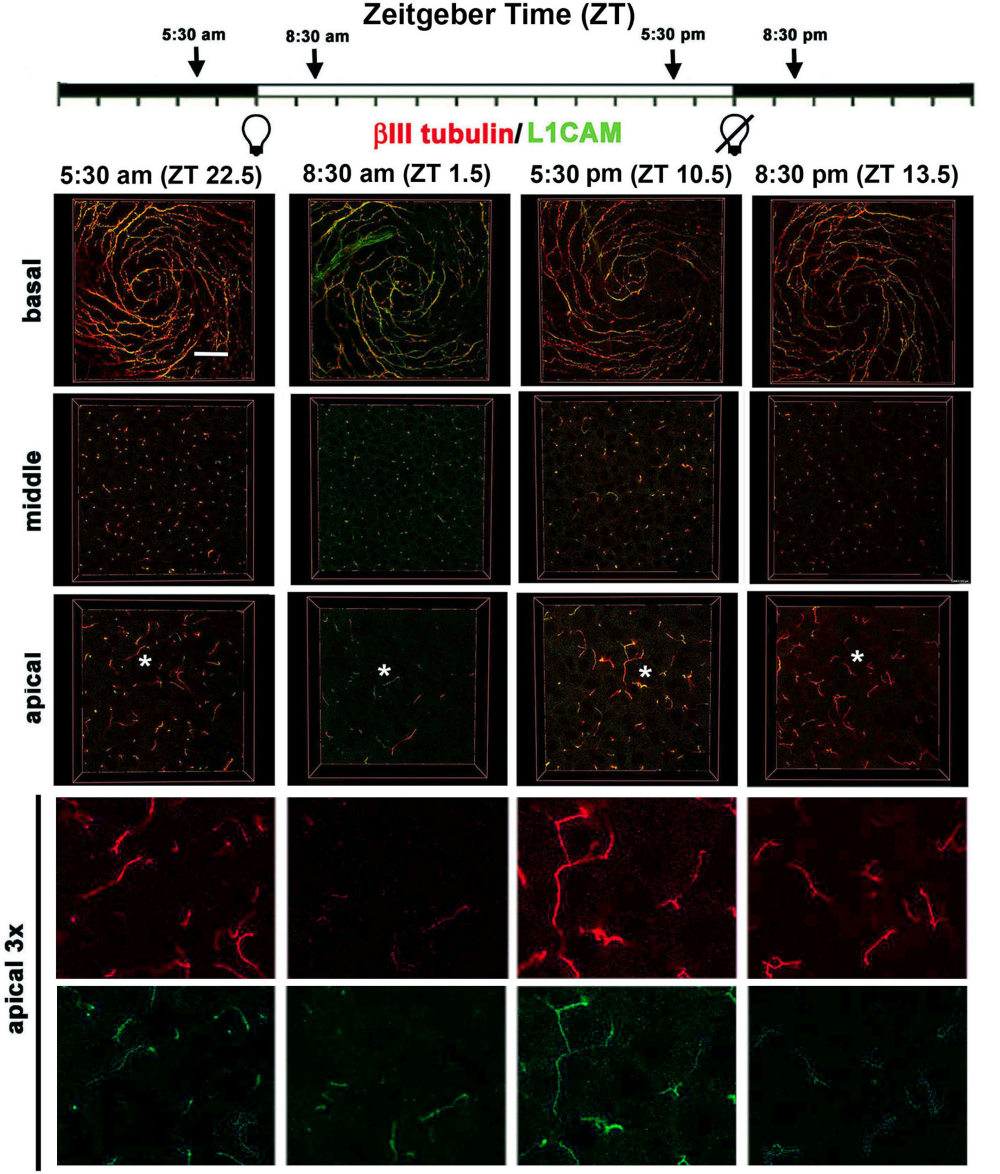
Localization of axonal proteins βIII tubulin and L1CAM in the mouse ICBNs and ICNTs visualized using 3D confocal imaging as a function of the time mice are euthanized. Four corneas from 2 male and 2 female mice euthanized at ZT 22.5, ZT 1.5, ZT 10.5, and ZT 13.5 were processed to visualize βIII tubulin and L1CAM. At the far right, the regions indicated by the asterisks in the apical most images are enlarged 3x with the two colors presented individually. These data are quantified in Figure 3. *Scale bar:* 25 µm

**Figure 3.**
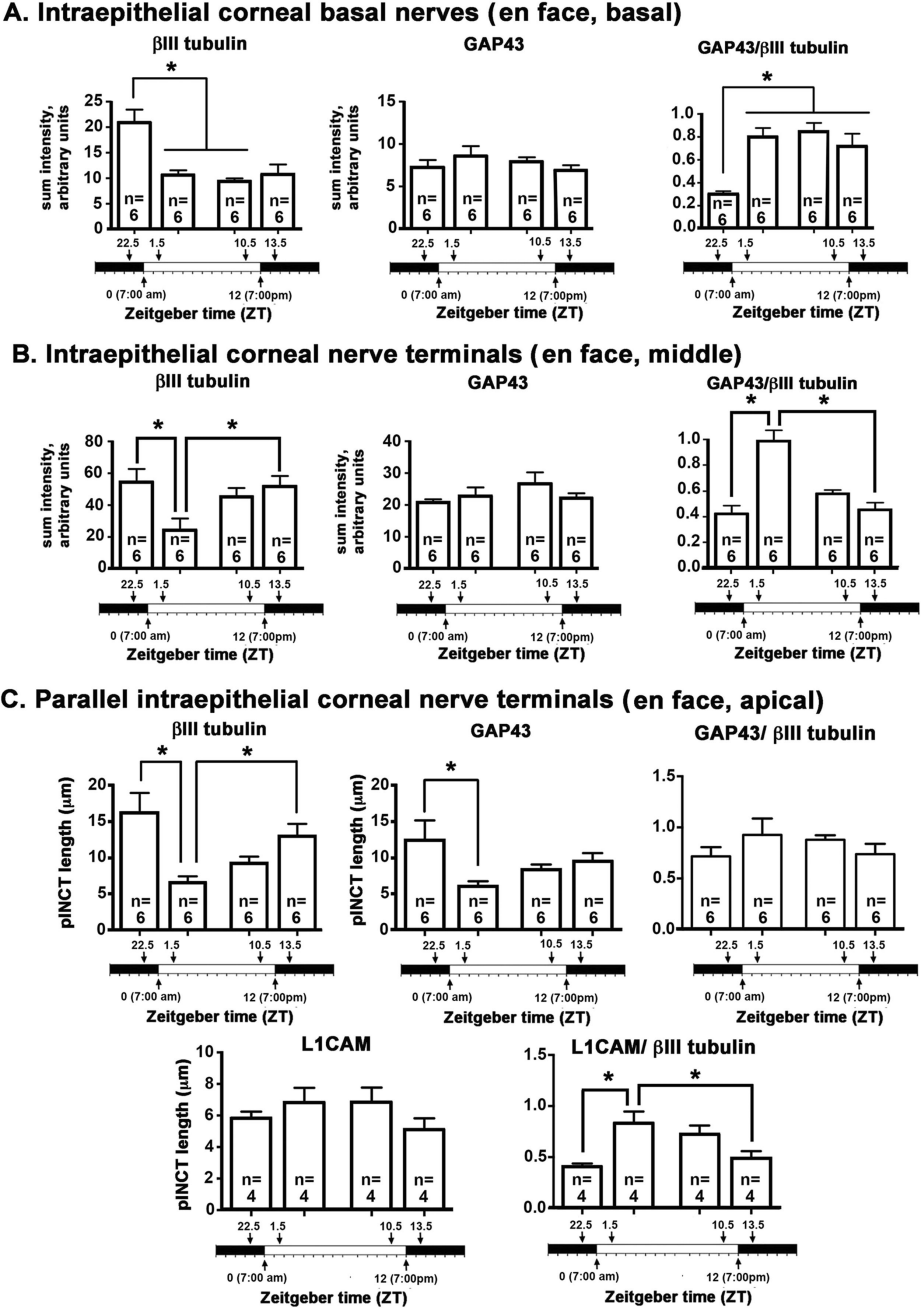
Changes in localization of βIII tubulin, GAP43, and L1CAM take place over time. **(A)** The expression of βIII tubulin in ICBNs is maximal 90 minutes before the time when lights are turned on at ZT 22.5; thereafter, βIII tubulin expression remains the same in the ICBNs. GAP43 expression in the ICBNs does not change significantly over time. The ratio of GAP43/βIII tubulin is lowest at ZT 22.5 and higher at all other time points indicating that axons are actively growing while the lights are on and continue to grow for 90 minutes after the lights are turned off at ZT 13.5. **(B)** ICNTs in the middle layers of the corneal epithelium appear as puncta. Significant differences are seen between ZT 22.5 and ZT 1.5 and between ZT 1.5 and ZT 13.5 for βIII tubulin. No significant differences are seen in GAP43; the ratio of GAP43/βIII tubulin in the ICNTs is highest at ZT 1.5. **(C)** In the apical aspect of the cornea where the ICNTs turn and assume a parallel orientation, there is a significant drop in the lengths of the pICNTs between ZT 22.5 and ZT 1.5 that is observed when the lengths of the pICNTs are quantified using βIII tubulin and GAP43; this is not observed when the lengths of the pICNTs are assessed using L1CAM.

**Figure 4.**
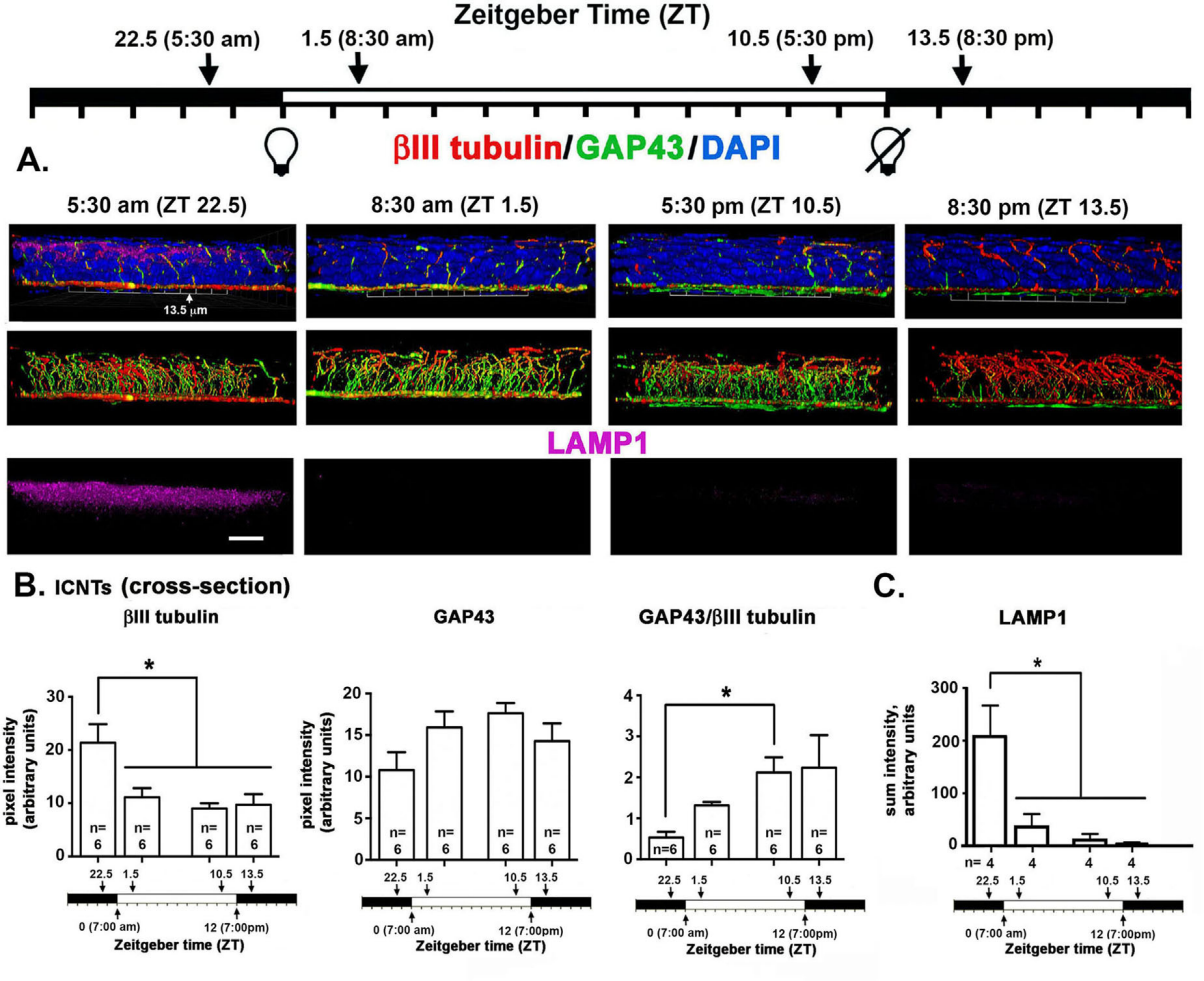
Localization of axonal proteins βIII tubulin, GAP43, and LAMP1 in the mouse visualized in cross-sections using 3D confocal imaging as a function of time mice are euthanized. **(A)** En face confocal image stacks through the corneal epithelium were obtained and rotated to generate a cross sectional view. The images project through 135 µm of cornea epithelium to reveal the ICNTs projecting apically and occasionally turning to generate pICNTs. While LAMP1 is expressed most abundantly in the apical most cell layers and appears most abundant at 5:30 am, the wide dynamic range of its expression complicated obtaining representative images. *Scale bar:* 20 µm. **(B)** We quantified βIII tubulin and GAP43 pixel intensity in the middle of the epithelium in the ICNTs in cross-sectional images. The expression of βIII tubulin in ICNTs decreases significantly between ZT 22.5 and ZT 1.5 and remains the same at ZT 1.5, ZT 10.5, and ZT 13.5. The ratio of GAP43/ III tubulin increased significantly between ZT 22.5 and ZT 10.5. (**C)** LAMP1 was quantified in five male and five female corneas. The expression of LAMP1 observed at ZT 22.5 was five- to sixfold higher than that seen at all other time points. By ZT 13.5, LAMP1 is at its lowest. By ZT 22.5, 90 minutes before the lights are turned on at ZT 0, LAMP1 expression is at its maximum.

**Figure 5.**
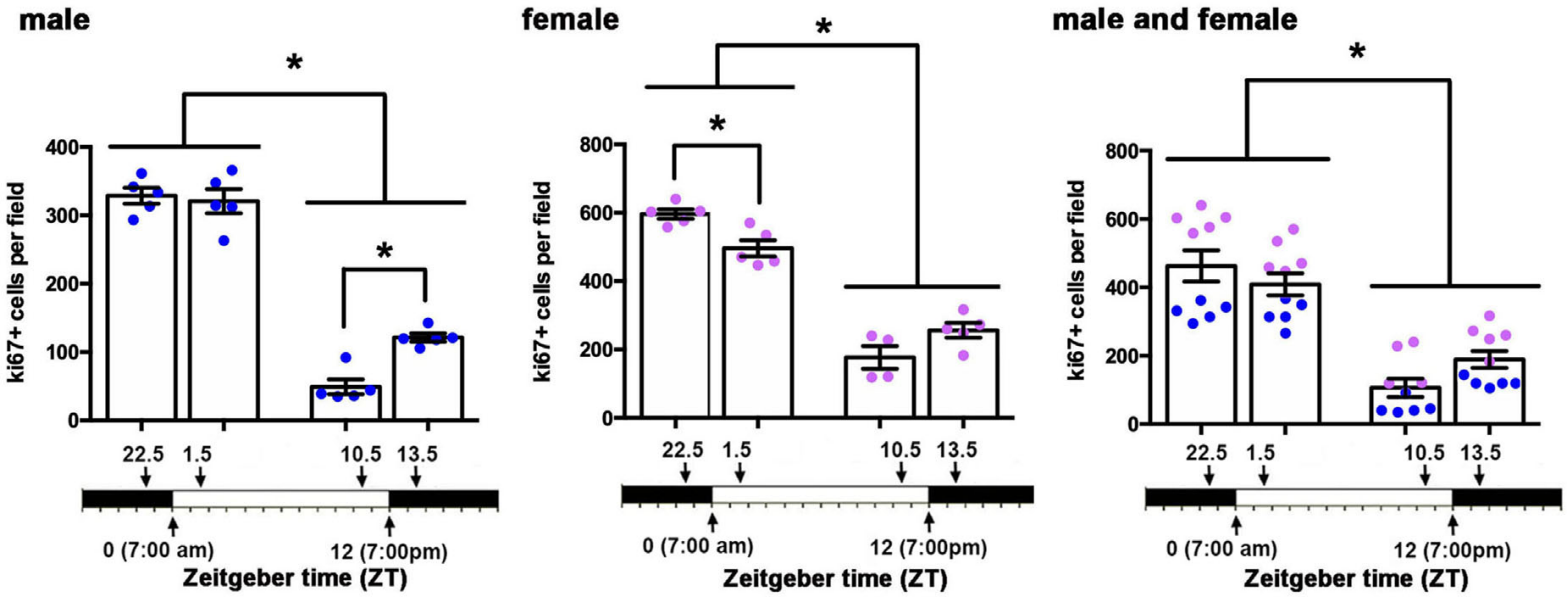
Corneal epithelial cell proliferation also varies as function of time. Corneal epithelial proliferation, measured as the numbers of ki67+ cells per field, was assessed in five male and five female corneas at ZT 22.5, ZT 1.5, ZT 10.5, and ZT 13.5. Epithelial proliferation is maximal at ZT22.5 and ZT 1.5 in males and females. Cell proliferation reduces dramatically at ZT 10.5 and ZT 13.5 before and after the lights are turned off in both sexes.

Confocal image stacks were obtained from five corneas for each time point and en face images showing βIII tubulin and GAP43 were generated at three sites within the epithelium (basal, middle, and apical). Representative en face images at the vortex near the center of corneas from male mice are shown for βIII tubulin and GAP43 in Figure 1 and for βIII tubulin and L1CAM in Figure 2. Quantification of data from n = 6 (three male and three female) corneas for GAP43 and βIII tubulin and n = 4 (two male and two female) corneas for L1CAM and βIII tubulin are presented in Figure 3. In addition, 3D confocal image stacks were rotated to yield cross-section projection views 135 µm long and 135 µm deep to allow us to appreciate how the ICNTs project apically. Representative cross-sectional images are shown in Figure 4, and their quantification from n = 6 (three male and three female) is shown in Figure 5.

The en face basal images presented in Figures 1 and 2 show the ICBNs. The vortex is well formed at all time points. As shown in Figure 1 and Figure 3A, ICBNs in the basal images express more βIII tubulin at ZT 22.5 (5.30 AM) which is 90 minutes before lights are turned on; this difference is significant when we compare ZT 22.5 to ZT 1.5 (8:30 AM) and ZT 10.5 (5:30 PM). GAP43 expression does not vary significantly over time. The ratio of GAP43/βIII tubulin in the ICBNs increases between ZT 22.5 and ZT 1.5 and remains elevated; these differences are significant and indicate that ICBNs are growing while the lights are on and continue growing until 90 minutes after lights are turned off at ZT 12. However, by ZT 22.5, growth has slowed significantly.

Although the basal images in Figures 1 and 2 show the ICBNs, the middle and apical images show the ICNTs that, in the middle images, appear as puncta indicating that the terminals are projecting apically and perpendicular to the epithelial basal cell-basement membrane. Quantification of βIII tubulin and GAP43 in ICNTs was also performed using single middle en face images, presented in Figure 1 with quantitation presented in Figure 3B and using cross-sectional images presented in Figure 4A with quantitation presented in Figure 4B. These analyses, performed on three male and three female corneas, show that there are also more βIII tubulin+ ICNTs at ZT 22.5 compared with all other time points. The differences seen in GAP43 expression were not significant and the GAP43/ βIII tubulin ratio increases significantly between ZT 22.5 and ZT 10.5 which is 90 minutes before lights go off. Thus, once lights turn on, although there are fewer ICNTs and ICBNs at the corneal center, the nerve terminals present are actively growing and continue growing for at least 90 minutes after lights are turned off.

### Axon Tips are Shed Soon After Lights are Turned on

In the apical most cell layers of the en face images in Figures 1 and 2 at ZT 22.5, ICNTs can be seen both as puncta and aligning parallel to the apical surface; we refer to the elongated apical terminals as parallel ICNTs (pICNTs). At ZT 1.5, we observed fewer and shorter pICNTs compared with all other time points; instead, we see puncta similar to those seen in images obtained from middle layers of the epithelium. We quantified the lengths of 15 to 20 GAP43+/βIII tubulin+ pICNTs per cornea in three male and three female corneas and the lengths of 15 to 20 L1CAM+/βIII tubulin+ pICNTs per cornea in two male and two female corneas from each time point using Image J. Data are presented in Figure 3C. The βIII tubulin data indicate that pICNTs are longest at ZT 22.5 (mean 17 µm) 90 minutes before lights are turned on and shortest at ZT 1.5 (mean 7 µm) 90 minutes after lights are turned on. Between ZT 1.5 and ZT 10.5, the pICNTs lengthen slightly to a mean of 9 µm, but the difference is not significant. By ZT 13.5, pICNT length is 12 µm, which is significantly longer than at ZT 1.5. The pICNT length continues to increase between ZT 13.5 and ZT 22.5, but the increase is not statistically significant.

Comparing the mean pICNT lengths determined using GAP43 and L1CAM to that obtained for βIII tubulin at each time point is revealing. Although pICNT length assessed using βIII tubulin is 17 µm at ZT 22.5, pICNT length assessed using GAP43 at the same time point is 12 µm and that of L1CAM is 6 µm; the expression of GAP43 and L1CAM relative to βΙΙΙ tubulin in pICNTs at ZT 22.5 is 72% and 40%, respectively. By contrast, at ZT 1.5, the expression of GAP43 and L1CAM relative to βΙΙΙ tubulin in pICNTs is 80% and 70%, respectively. This indicates an increase in L1CAM localization on growing nerve terminals after lights are turned on. By ZT 10.5, the expression of GAP43 and L1CAM relative to βΙΙΙ tubulin in pICNTs is 70% and 50% respectively, and by ZT 13.5, the expression of GAP43 and L1CAM relative to βΙΙΙ tubulin in pICNTs is 79% and 46%, respectively. These data indicate that before pICNTs are shed, their relative expression of L1CAM is low compared with βIII tubulin; by contrast, when pICNTs are regenerating at ZT 1.5 and ZT 10.5, the normalized expression of L1CAM in pICNTs is high (70% and 50%, respectively). At ZT 13.5 after lights are turned off, the normalized expression of L1CAM continues to drop to levels similar to those at ZT 22.5. These data indicate that the severing of the apical tips of the nerve terminals takes place when the relative expression of L1CAM localization on the nerve terminals is reduced to βIII tubulin, whereas the relative expression of L1CAM is high during the time when nerve terminal growth is maximal.

In addition to showing the localization of βIII tubulin and GAP43, the cross-sectional images in Figure 4 also show intense expression of LAMP1 in suprabasal cells at ZT 22.5. Although corneal basal epithelial cells also have lysosomes, lysosomal biogenesis is increased in differentiating cells of the epithelium where lysosomes function in autophagy to degrade organelles in cells before the cells are sloughed off.^28^ The large dynamic range of expression in LAMP1 made it difficult to quantify changes in LAMP1 expression over time. By analyzing LAMP1 in n = 5 male and n = 5 female corneas, we could confirm that LAMP1 is expressed at significantly higher levels at ZT 22.5 than at any other time point (Fig. 4C). At ZT 1.5, ZT 10.5, and ZT 13.5 images obtained at the same laser settings used for ZT 22.5 often show no LAMP1 as indicated by the representative images shown. Yet, in a minority of the images, Image J detected low levels in several of the 10 images obtained at each time point as indicated in the bar graphs shown.

### Corneal Epithelial Cell Proliferation and Thickness Vary as a Function of Time

Cell proliferation and/or increased desquamation may both contribute to the reduction in ICBNs and shedding of the pICNTs seen between ZT 22.5 and ZT 1.5. There is a limited surface area on the corneal epithelial basement membrane to accommodate corneal epithelial cells. Proliferation of basal cells leads to the displacement of differentiating cells from the basal cell layer into the suprabasal or wing cell layer, eventually leading to the sloughing off of the apical most squames. To get a better understanding of the impact of cell proliferation and desquamation on the ICNs, we next assessed corneal epithelial cell proliferation by counting the number of ki67+ cells per field in en face flat mounted mouse corneas at both the center and periphery in 5 male and 5 female corneas from mice sacrificed at ZT 22.5, ZT 1.5, ZT 10.5, and ZT13.5. Data are presented in Figure 5. In males, cell proliferation was maximal and the same at ZT 22.5 and ZT 1.5; it is six times lower at ZT 10.5 and three times lower at ZT 13.5. In females, cell proliferation was significantly higher at ZT 22.5 compared to ZT 1.5 and higher at both ZT 22.5 and ZT 1.5 than at ZT 10.5 and ZT 13.5. The PM time points were three times lower than the AM time points. Compared to males, corneal epithelial cell proliferation was significantly higher in female corneas at ZT 22.5 and ZT 1.5 compared to ZT 10.5 and ZT 13.5. Despite elevated overall cell proliferation in female mice, the changes over time between sexes were similar; combining corneal epithelial cell proliferation data from male and female corneas shows that cell proliferation is maximal 90 minutes before and 90 minutes after lights go on and drops threefold over a 12-hour time period. The loss of ICBNs and pICNTs seen at ZT 1.5 occurs when cell proliferation is maximal.

**Figure 6.**
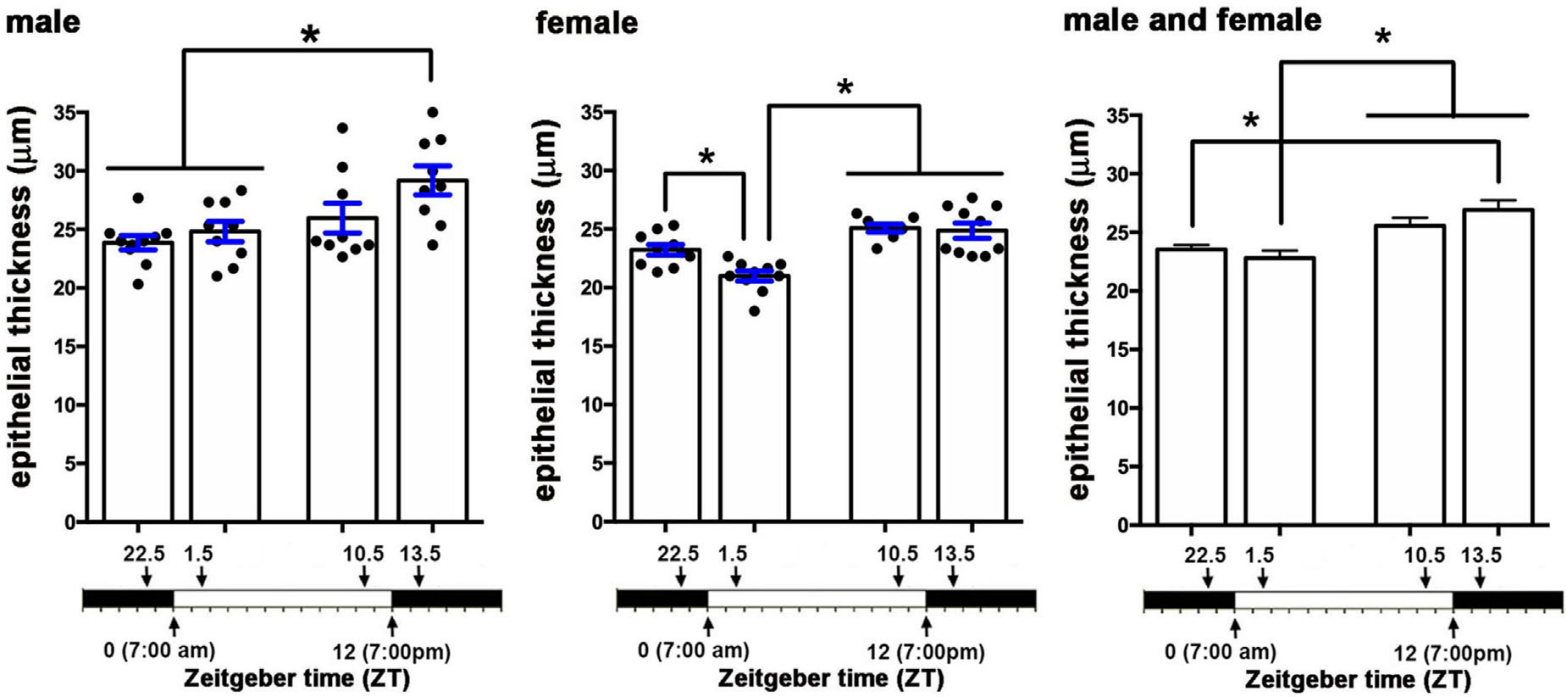
Corneal epithelial thickness varies as function of time. Corneal epithelial thickness, measured in fixed corneas, was assessed in 10 male and 10 female corneas at ZT 22.5, ZT 1.5, ZT 10.5, and ZT 13.5. Epithelial thickness is maximal at ZT 13.5 in males and at ZT 10.5 and ZT 13.5 in females. Before and after the lights are turned on at ZT 22.5 and ZT 1.5, epithelial thickness is reduced. These data are similar in both male and female mice.

We next assessed the thickness of the corneal epithelium in male and female mice to determine whether we could also observe changes over time; data were obtained using confocal imaging to measure corneal epithelial thickness in en face corneas and are presented in Figure 6. We counted the number of 0.5 µm sections needed to move from the apical squames to the basal aspect of the basal cells in five male and five female corneas. In male mice, the epithelium was significantly thinner at ZT 22.5 and ZT 1.5 compared to ZT 13.5. In female mice, the epithelium was also significantly thinner at ZT 1.5 compared to ZT 22.5; in addition, it was thicker at ZT 10.5 and ZT 13.5. When the data for epithelial thickness from male and female corneas are combined, there is a significant increase in epithelial thickness between ZT 1.5 compared to ZT 10.5 and ZT 13.5. Epithelial thickness increases over time when lights are on. Between ZT 13.5 and ZT 22.5, epithelial thickness decreases significantly. In the combined data from male and female corneas, there are no significant differences in epithelial thickness 90 minutes before and 90 minutes after the lights are turned on.

### Corneal Epithelial Cell Apical Squames are Shed at a Greater Rate Prior to when Lights are Turned on

**Figure 7.**
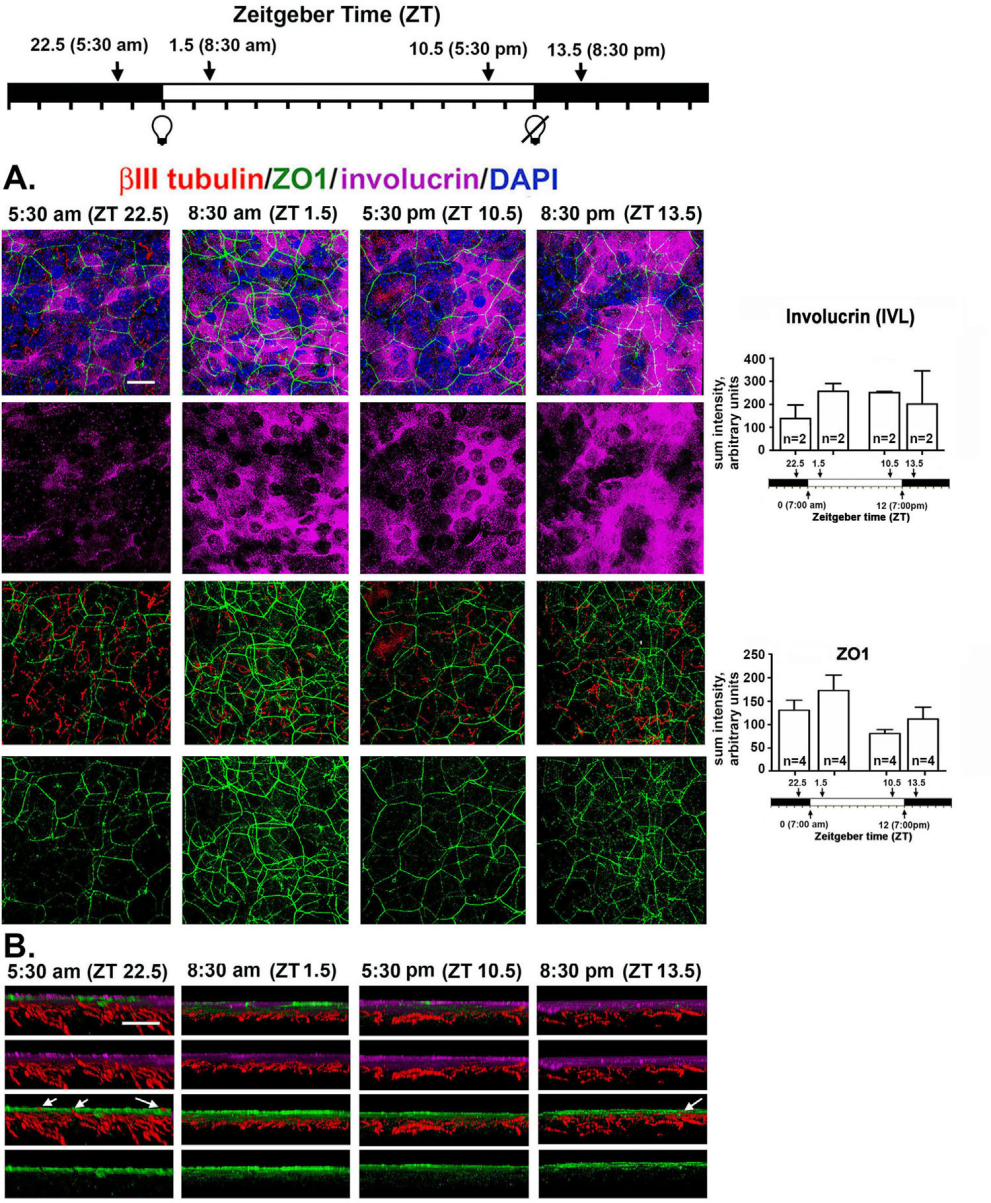
Localization and expression of tight junction protein ZO1 and terminal differentiation protein IVL are altered 90 minutes before lights are turned on. **(A)** Shown are representative en face confocal images projecting through the apical most 10 µm of the cornea that was stained to show βIII tubulin (*red*), ZO1 (*green*), involucrin (*magenta*), and DAPI (*blue*). ZO1 is expressed at cell/cell borders at ZT 22.5 but areas are seen where it is disrupted. While at all other time points ZO1 is expressed uniformly in all apical cells, by ZT 13.5, expression is reduced. While the cytoplasm of the majority of the apical cells contain involucrin, there are more cells that do not express detectible IVL at ZT 22.5 compared to all other time points. *Scale bar* for en face image **A:** 25 µm; (**B)** The 3D cross-sections projecting through 135 µm of the tissue were generated showing the apical 10 µm of the cornea. The involucrin+ cells are also surrounded by ZO1+ cell membranes and at ZT 22.5, several ICNTs insert between the apical squames (*arrows*). By contrast, at ZT 1.5 ICNTs do not penetrate the apical cell layer**.** At ZT 13.5, a single ICNT is seen that penetrates all the way to the apical cell layer. *Scale bar* for cross-sectional image **B**: 10 µm.

The reduction in epithelial thickness seen at ZT 22.5 and ZT 1.5 could arise due to an increase in desquamation rate before those time points. An increased rate of displacement of the apical cells could contribute to the loss of pICNTs seen between ZT 22.5 and ZT 1.5. To obtain additional insight into the role desquamation plays in the loss of pICNTs, we next stained corneas with antibodies against βIII tubulin, Zonula Occludens-1 (ZO1), and involucrin (IVL); representative en face images are shown in Figure 7A. To eliminate staining of the ICBNs to focus on the ICNTs in the apical cell layers, 3D confocal projection images are generated through the apical 10 µm of the cornea at each time point.

IVL is one of 61 proteins that comprise the epithelial differentiation complex and is expressed in differentiating cells in the apical most cell layers of the skin and cornea.^29^ At ZT 22.5 before the lights go on, IVL localization within the apical-most epithelial cells is restricted to subsets of cells where it is expressed diffusely. While lights are on and for 90 minutes after the lights are turned off at ZT 13.5, IVL is expressed abundantly in the majority of the apical cells. Although images suggest altered localization and expression of IVL over time, when quantified, the differences in IVL expression observed were not significant.

Tight junctions (TJs) containing ZO-1 form physical barriers that prevent loss of water and penetration of the epithelium by microorganisms. In stratified squamous epithelia like the epidermis and cornea, TJs are present at the cell: cell borders of the apical most squames.^30^^,^^31^ When apical squames are shed, ZO1 localization is disrupted; in response to desquamation, underlying epithelial cells up-regulate not only IVL, but also ZO1. As shown in Figure 7A, ZO1 is present at cell: cell borders and, at ZT 22.5, is patchy; after lights go on, ZO1 localization at cell: cell borders increases and becomes more uniform across the apical surface. By ZT 13.5, ZO1 expression appears to decrease. Like IVL, the differences in expression of ZO1 seen were not significant. The changes in both IVL and ZO1 localization between ZT 22.5 and ZT 1.5 indicate that an increase in shedding of the apical epithelial cells takes place around ZT 22.5.

To visualize the relationships between the IVL+ZO1+ apical cells and the ICNTs, we also generated cross-sectional images that project through 135 µm of the apical 10 µm of the mouse cornea; representative images are shown in Figure 7B. At ZT 22.5, ICNTs and pICNTs project into the apical most cell layer where IVL and ZO1 are expressed as shown by the arrows in Figure 7B. At ZT 1.5, none of the ICNTs extend into the apical most squames. As time progresses while lights are on, the ICNTs continue to grow and extend apically; by ZT 13.5, one is seen extending into the apical most cell layer as shown by the arrow.

Although data show evidence for the parallel tips of the ICNTs being shed after ZT 22.5, LAMP1, IVL, and ZO1 expression indicate that the rate of desquamation is greater at ZT 22.5 when pICNTs are at their longest. Cell proliferation is high at ZT 22.5 and remains high at ZT 1.5 when pICNTs have been shed. Thus, whereas elevated cell proliferation is more closely linked to the shedding of the nerve terminals than desquamation, the fact that the rise in desquamation occurs before nerve terminals are shed indicates that both events play roles in the regulation of axon shedding and growth. Regrowth of ICNTs slows after lights are turned off but does not stop, resulting in pICNTs being their longest at ZT 22.5, a time point when the pICNTs also have the least amount of L1CAM on their surface.

### Loss of ICNTs Also Leads to a Reduction in ICN Density as Assessed by Sholl Analysis

**Figure 8.**
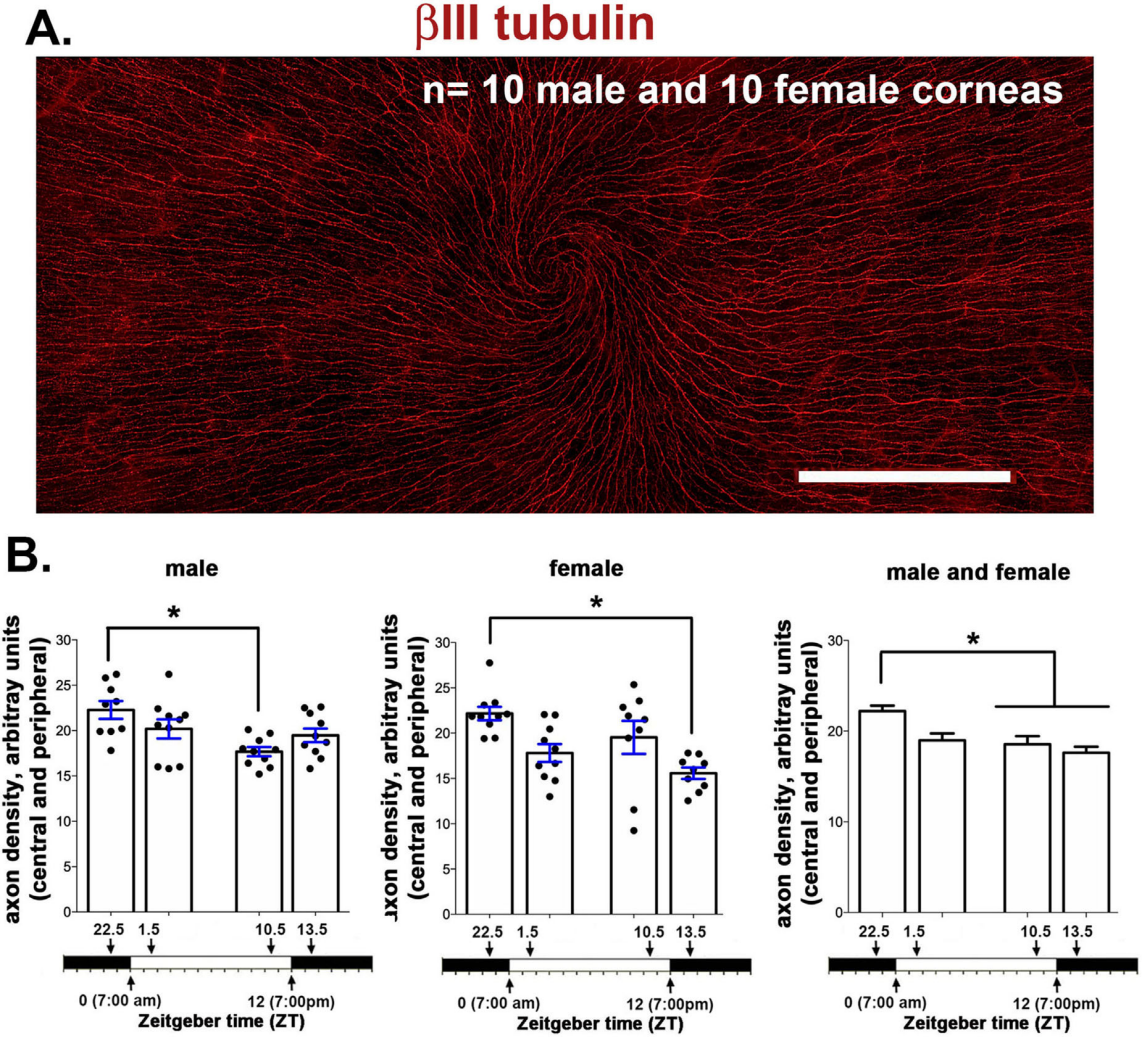
Axon density, assessed by Sholl analysis, also varies as function of time. (**A)** Representative image taken of a female cornea from a mouse sacrificed at ZT 10.5. The cornea was stained with antibodies against βIII tubulin to reveal the ICNs. Each cornea was imaged using a spinning disk microscope with 7 × 3 individual images stitched together as described in the methods section. *Scale bar:* 500 µm. (**B)** Axon density was assessed in 10 male and 10 female mice at ZT 22.5, ZT 1.5, ZT 10.5, and ZT 13.5 using Sholl analysis. The data shown were obtained by assessing axon density at four sites at the corneal periphery and three sites in the corneal center for males and females and after combining data from both sexes. Axon density is maximal at ZT 22.5 and lower 90 min before and 90 min after lights are turned off. In both sexes, axon density is greatest at ZT 22.5 and lowest between ZT 1.5 and ZT 13.5.

Data presented above show that pICNTs are shed between ZT 22.5 and ZT 1.5; severing corneal axons leads to transient retraction and degeneration of proximal axons.^15^ βIII tubulin expression in the en face basal images is reduced 90 minutes after lights are turned on. The high-resolution 3D confocal imaging data presented above were obtained from 135 µm × 135 µm sites at the vortex near the corneal center of six mice (three male and three female). To determine whether axon density is reduced over the entire corneal surface as a function of time, Sholl analysis was performed in 10 male and 10 female corneas, and data are presented in Figure 8. As described previously,^18^ Sholl analysis assesses axon density using en face projection images generated by flattening 3D spinning disk confocal images. Axon density is quantified over a larger surface area (2.1 mm × 0.9 mm). In both males and females, axon density is maximal at ZT 22.5. In males, axon density is lowest at ZT 10.5; in females, it is lowest at ZT 13.5. When we combine data from males and females, axon density is significantly higher at ZT 22.5 compared with ZT 10.5 and ZT 13.5. Axon density decreases between ZT 22.5 and ZT 1.5, but the difference is not statistically significant when all four times points are evaluated by ANOVA. The time-dependent reduction in ICBNs and the shedding of the ICNTs seen between ZT 22.5 and ZT 1.5 represent an overall reduction in axon density on the cornea.

## Discussion

**Figure 9.**
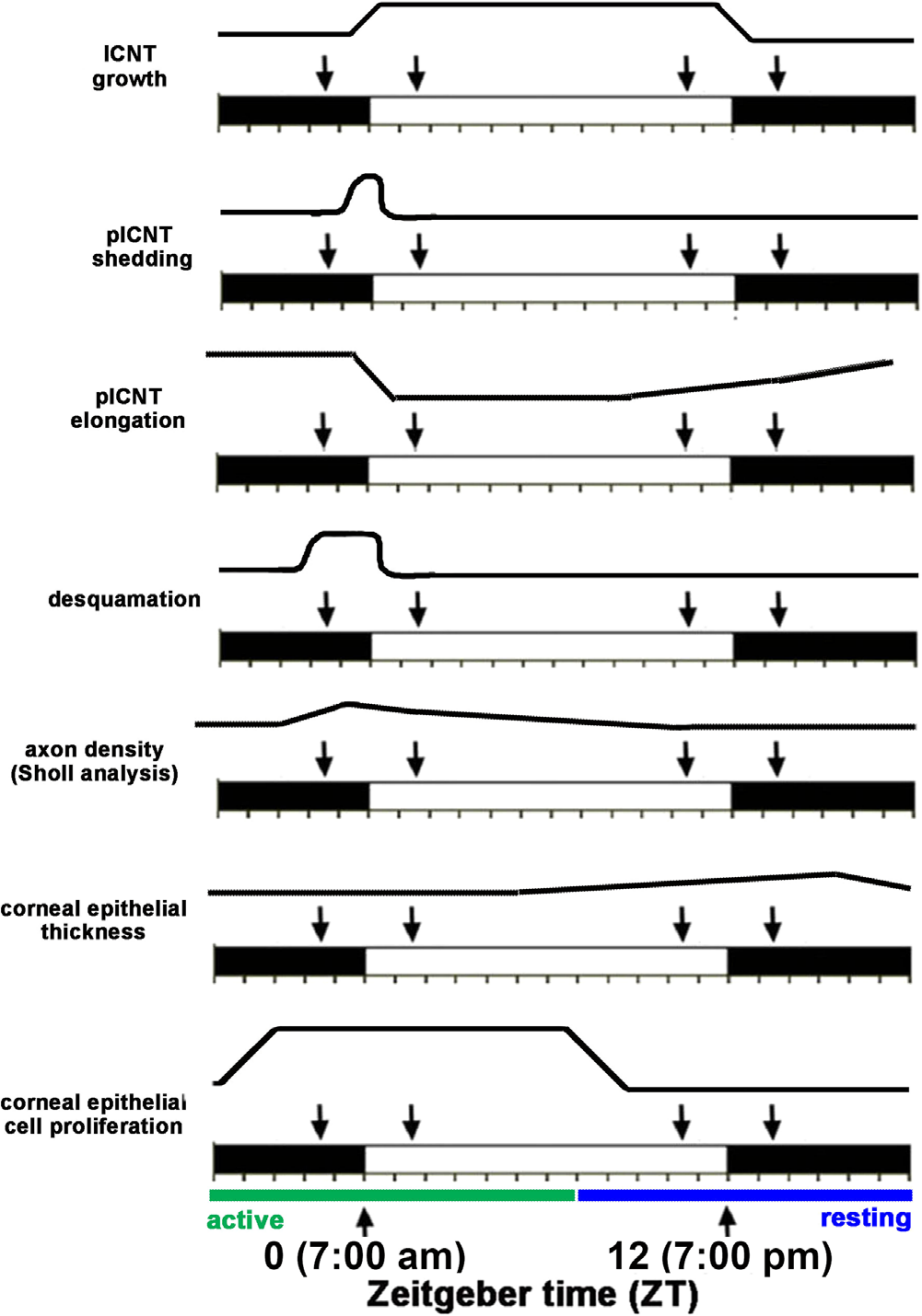
Summary of the changes in the mouse cornea and its nerves that vary over time. The Zeitgeber scale is shown. The *white area* indicates when lights are on and the black area when lights are turned off. In these studies, lights are turned on at ZT 0 and off at ZT 12. We evaluated corneas 90 minutes before and 90 minutes after lights were turned on and off; those time points are indicated by the *arrows*.

Here we show that the light induced shedding of the corneal sensory nerve terminals is under diurnal control with the timing similar to the shedding of the outer segments of the photoreceptors. Because we did not maintain a cohort of mice in constant darkness to compare results to the changes reported here, we cannot claim that the changes we report are under circadian control. Figure 9 shows a schematic summarizing the changes seen in the ICNs and the corneal epithelial cells as a function of the time at which mice are euthanized. These data indicate that the clock that controls the shedding of the photoreceptor outer segments one to two hours after lights are turned on may also control the shedding of the tips of the intraepithelial corneal nerve terminals in the cornea.

Data presented here show that axon shedding of the parallel intraepithelial corneal nerve terminals is maximal 90 minutes after lights are tuned on, consistent with the upregulation of shedding and phagocytosis of the outer segments. These events occur at times when cell proliferation is elevated and following an increase in desquamation seen at ZT 22.5; LAMP1, a lysosomal marker whose expression is elevated during autophagy^32^ and in epithelial cells during terminal differentiation in stratified squamous epithelia^33^, is expressed at higher levels within the apical cell layers of the corneal epithelium at ZT 22.5 compared to other time points. ZO1 localizes exclusively to tight junctions in the stratified squamous epithelium.^34^ IVL is also localized within the apical most epithelial cells. As the apical most squames are shed during terminal differentiation, underlying cells up regulate ZO1 and IVL to maintain the tight junctional barrier and differentiation state of the epithelial cells.

Before the time when lights are turned on, cell proliferation rate increases. It is high 90 minutes before (ZT 22.5) and 90 minutes after (ZT 1.5) lights are turned on; cell proliferation dropped fourfold 90 minutes before (ZT 10.5) and after (ZT 13.5) lights are turned off. A recent study by Xue and colleagues^10^ showed that C57BL6 mouse corneal epithelial cells had elevated cell proliferation between ZT 19 and ZT 7, a period they refer to as the *active period*; cell proliferation dropped between ZT 8 and ZT 18, a period they refer to as the *resting period*. Our cell proliferation data, obtained using BALB/c mice, confirm and extend those data to another mouse strain. Axon tip shedding takes place within a 180-minute time period (after ZT 22.5 and before ZT 1.5) when cell proliferation remains high. LAMP1 expression is highest and ZO1 is discontinuous 90 minutes before lights are turned on (ZT 22.5), indicating that desquamation has already initiated before lights are turned on and ICNTs are shed.

We assessed epithelial thickness in fixed corneas processed for whole mount staining using the confocal microscope to move through the epithelium in 0.5-µm layers. While all of the corneas were intact and unwounded in these studies, the changes we report in epithelial thickness could be due to changes in the epithelial barrier over time which permits more or less swelling during post-enucleation processing of the eyes. The barrier would be expected to be impaired most during periods of most active desquamation where ZO1 localization is both reduced and less uniform, when few cells express IVL, and LAMP1 expression is elevated; these events are documented to occur at ZT 22.5. Yet, epithelial thickness is less at that time point and is maximal at time points where pICNTs are actively elongating and desquamation is not taking place.

Our data show that during the active period, which begins before lights are turned on and ends before lights are turned off, more is happening in the cornea than just elevated cell proliferation: shedding of axon tips, increase in ICN density (ICBN, ICNT, and pICNT branching and elongation), and desquamation. Early in the resting period, epithelial thickness increases. This presumably is due to the increased numbers of cells generated by cell proliferation during the active period. As the resting period continues, epithelial thickness decreases.

Circadian clocks exist at several levels in all organisms. In mammals, intrinsically photosensitive retinal ganglion cells (ipRGCs) express a pigment protein called melanopsin (OPN4).^35^^,^^36^ OPN4 is a member of a subfamily of opsins which includes encephalopsin (OPN3) and neuropsin (OPN5); the roles played by OPN3, OPN 4, and OPN5 in regulating the circadian clock in the numerous cell types present in the eye have only recently been investigated.^9^^,^^37^ Genetically engineered mice lacking OPN4 in their ipRGCs show reduced light aversion and corneal sensitivity after treating the ocular surface with the irritant benzalkonium chloride.^38^ These studies suggest that loss of circadian clocks impacts corneal epithelial homeostasis.

Studies of aging^39^ show the negative impacts of aging are accompanied by a decline in the functioning of circadian clocks. We have shown that mice lose ICNs and corneal sensitivity as they age.^40^ The studies presented here were performed on young male and female mice seven to nine weeks of age; our data suggest that aged mice will show alterations in ICNT shedding and regrowth over time during the day.

Mice are nocturnal animals whose activity level increases in the dark compared to the light. Although humans are diurnal, many of the changes in circadian gene expression observed in the retinas and corneas of mice occur in the human eye. This is true for the shedding of photoreceptor outer segments which occurs 1-2 hours after eyelid opening in humans and when lights are turned on in humans and mice.^35^^,^^36^ If the tips of the sensory nerves are shed in the human cornea soon after eyelid opening, it might be expected to lead to an increase in ocular discomfort early in the morning compared to late at night. A study to assess diurnal variations in signs and symptoms in 21 patients with dry eye symptoms showed that corneal discomfort was greatest in the morning whereas corneal sensitivity was greater in the evening.^41^ Although a small human study, these data are consistent with increased ocular pain after ICNT shedding in the morning and increased sensitivity due to the increased lengths of the parallel ICNTs in the evening. Human corneal epithelial thickness increases overnight while eyelids are closed.^7^^,^^42^ After eyes open, epithelial thickness quickly returns to its baseline and is maintained throughout the day. Mice sleep during the day and keep their eyes closed more despite lights being on. We found that the mouse corneal epithelium is thicker in the evening around the time when lights are turned off, and mice increase their activity and open their eyes more.

In the mouse cornea, parallel nerve terminals are shed at around the time lights are turned on. Like the RPE, corneal epithelial cells must increase their phagocytosis of this debris to prevent it from accumulating within the tissue. Although there is likely a low level of constitutive shedding of the sensory axon tips, the only time we observed parallel ICNTs decrease in length was between ZT 22.5 and ZT 1.5. At all other time points they appear to be elongating. When axons are severed by trephine injury, the proximal axon stubs that remain attached to the trigeminal neuron “retract” for several hours before beginning to reinnervate the site denervated by injury. Sholl analysis, which provides an assessment of ICBN density, shows that overall axon density also decreases after the axon tips are shed at ZT 1.5. Although corneal epithelial cells stop proliferating before the time when lights are turned off, parallel ICNTs continue to elongate in darkness. These data suggest differences in corneal sensitivity and pain sensation over time in the mouse. Further studies are needed to address this issue. The changes we report here have important implications for the interpretation of results from wound healing studies and studies testing the efficacy of various treatments. A better understanding of ocular pain and discomfort in the cornea may be achieved by applying knowledge from studies of outer segment shedding and RPE phagocytosis.
